# Supplementary material for: Surveillance of Salmonella enterica serovar Typhi in Colombia, 2012–2015
Source: PLoS Negl Trop Dis. 2020 Mar 10;14(3):e0008040. doi: 10.1371/journal.pntd.0008040 (PMC7083327; doi:10.1371/journal.pntd.0008040)
Supplement: Attachment — Submitted filename: S.Typhi Colombia_020120_CL.docx [file pntd.0008040.s004.docx]

Professor Stephen Baker

sbaker@oucru.org

3^rd^ January 2020

Re: Submission to PLoS NTDs

Dear Editor

We wish the modified manuscript “**Surveillance of *Salmonella enterica serovar* Typhi in Colombia, 2012-2015**” by Paula Diaz-Guevara and other authors to be considered for publication in PLoS NTDs. Given the subject matter with think that PLoS NTD is the most suitable journal in which to communicate our findings; this article has not been submitted elsewhere. We thank you and the reviewers for their efforts, and we think we have addressed all of the reviewer’s comments in the revised article, these modifications are outlined below.

**Methods**

Reviewer #2: 

1. Line 130. It is mentioned that certain areas of the country have strengthened their surveillance for typhoid fever. If those specific areas or departments can be cited that would be helpful.

*The whole country benefitted from the increased surveillance, but this is difficult to quantify, therefore we have removed this so it isn’t misleading.*

2. Line 133: Defining “non-endemic” as “incidences lower than the national average” is not the usual usage and meaning of “endemicity”. Endemicity repetitive, predictable occurrence over time rather than relative incidence (incidence against a national average). Characterizing all regions below the national average as non-endemic could lead to under-reporting of endemicity and over-reporting of sporadic transmission. The U.S. CDC and W.H.O / P.A.H.O. have definitions of sporadic, endemic, hyperendemic transmission; as well as epidemic, outbreak, cluster, pandemic, etc.

*The term endemic has been replaced in the title and non-endemic has been replaced in the methods*

--------------------

**Results**

Reviewer #1: The analysis matches the plan and the results are clear and clearly presented. I found the maps to be a little blurred.

*The figures have been remade where required*

Reviewer #2: 1. Of 468 S. Typhi isolates, 402 came from seven departments. The department of Norte de Santander accounted for 190 (47.3%) of these 402 isolates. This result immediately catches the attention of the reader.

*This potential bias has been noted in the discussion*

2. Line 193-194, the statement “almost half of cases were affiliated to the national contributory health insurance scheme” is explained. If the authors are implying that this impacts surveillance, they should elaborate on this point. Also, “insurance scheme” is not discussed elsewhere, except itemized under Table 1. 

*A comment on this has been made in the discussion, but not expanded*

3. Figure 1 should explain the quantitative significance of the dot size in the legend and/or caption. If the dot sizes have no absolute meaning and are listed in steadily decreasing order based on PFGE type prevalence, perhaps to assist in differentiation by both size and color, then that must be stated so that misinterpretation is avoided. Currently, it might be interpreted that one dot of -0115 represents a prevalence 25-times greater than one dot of -0155 (roughly gauging relative dot size). Also, does each dot represent a hospital/reporting site or a patient’s home coordinates?

*Figure one has been modified, the dots are now the same size and numbers added to the legend for clarity.*

4. Lines 205-206. Does Sivigila define “endemic”?

*Sivigila uses WHO/OPS definitions, this has been clarified*

5. The first paragraphs of the Results discuss the number of cases by department and smaller administrative units. A table that presents: the mean annual population of each of these seven departments during the years 2012-2015 (derived from either the 2005 or 2018 census data); the mean number of S. Typhi cases over 2012-2015 and the mean annual incidence per 100,000 persons for those years, would be very helpful.

*This has been added in Supp Table 2*

Some departments, such as Norte de Santander, that have been the site of extensive legal and illegal immigration from Venezuela during 2014 and 2015 may have notable numbers of high-risk persons who are not reflected in the 2018 census. Nevertheless, whatever denominators are available should be used to calculate incidence rates. Then some of the figures can be modified to have spot maps based on incidence rather than just number of cases, should be crafted. 

*Figure 1 has been modified so that it now reflects both minimum incidence per department and location of the distinct pulsotypes*

6. Line 234: Table 3 does not represent the result that is stated immediately prior and in reference to the table.

*We have moved the reference from table 3 into the text*

7. Line 244-245: The sentence, “Notable, Group III contained the majority of organisms […] and nalidixic acid (n=10)” is misleading because Group III also contained 356 out of 402 characterized isolates. A percentage/proportion might be included in parentheses and a statistical test could be performed to support this association, if it is to be deemed “notable.” It should also be further discussed in the discussion section.

*We agree this was misleading and we have removed the offending comment*

8. Figure 2: The four maps are inconsistent with the text (lines 236-247) and the figure 2 caption. See below. 

***Lines 236-247:*** Group 1 has 13 isolates in 7 departments. Group 2 has 27 isolates in 6 departments. Group 3 has 356 isolates in 14 departments. Group 4 has 6 isolates in 4 departments.

*The text has been changed to the right numbers.* *To be clearer the dates of PFGE cluster were added to Table S1*

There are several inconsistencies (numbers and colors) that need addressing. 
The data contained within lines 265-274 could be better understood and discussed in a table or figure. 

*The inconsistences of (numbers and colors) have been corrected in the legend of Figure 2*.

Lines 287-288: What are the “three most common Colombian pulsotypes” specifically according to Figure 5? They are not clearly labeled or identified, and the labels in Figure 5 do not match the labels in Figure 1 or Table 3.

*Only the Colombian pulsotypes that matched a regional pattern in other countries are shown in Figure 5. For clarity the national pattern ID has been added.*

9. It was not possible to download reference #22.

*These have been checked*

10. Line 197: “liquid corporal-secretions” should be changed to “normally sterile body fluids”.

*This has been edited*

11. Figure 1 caption – “Kerner” seems to be a misspelling of “Kernel”.

*This is no longer required*

12. Figure 3 requires a scale on the map and again, “Kerner” seems to be a misspelling of “Kernel”.

*This has been edited*

13. Figure 4 caption: Lists cases by municipality, but the maps in Figure 4 do not label these municipalities.

*Figure 4 does not comment on municipality; however, these data can be found in Table S1*

14. Lines 276-288 “Colombian Salmonella Typhi in a Latin America framework”. In order to assess the relevance of this analysis the readers need to know how representative the isolates are for the other countries and how these isolates were obtained. Without this information it is hard to judge the relevance of comparing a few isolates from other countries in South America.

*This information has been added where required*

Reviewer #2: 1. Line 300. “We identified a particularly high burden of typhoid in Cucutá”, the capital city of Norte de Santander department. This city has been an epicenter of Venezuelan migration. The isolates from Cucutá should be compared to Venezuelan isolates of S. Typhi, if the authors have some. 

*Due to the ongoing situation in Venezuela we were unable to access new isolates for genotyping*

2. Do the authors have any way to differentiate which isolates came from Venezuelan immigrants and refugees? Minus the isolates from this city and this department, the total Colombia burden drops almost in half. So it is critical to attempt to ascertain how many of the typhoid cases derive from the Venezuelan refugee crisis.

*This is almost impossible to define due to the limitations of the data collection but have commented on this in the discussion*

5. Lines 341-358. To reiterate, without knowing more about the isolates from the other Latin American countries, including how they were selected, it is difficult for the reader to accept categorically the conclusions drawn by the authors. More information about these strains and softening of statements would strengthen the paper.

*This has been edited as suggested*

--------------------

**Editorial and Data Presentation Modifications?**


Use this section for editorial suggestions as well as relatively minor modifications of existing data that would enhance clarity. If the only modifications needed are minor and/or editorial, you may wish to recommend “Minor Revision” or “Accept”. 

Reviewer #1: Line 126: sentence does not make sense. Do you mean ’Cases were defined as those with …’?

*This has been edited*

Line 131: Not sure what reiterative epidemiological behavior is. Could this be clearer?

*This has been edited for clarity*

Line 197: What are liquid corporal-secretions?

*Normally sterile body fluids; this has been edited*

Line 203 and line 205: How does the public health department define outbreaks, endemic and non-endemic?

*The national surveillance guidelines define an outbreak as the unusual increase in the number of cases, two or more epidemiologically related cases, or the sudden appearance and dissemination of disease located in a specific location. Endemic is the constant presence and /or usual prevalence of a disease or infectious agent in a population within a geographic area. Non-endemic is the lack of a disease or infectious agent in a population within a geographic area, apart from occasion reported cases or outbreaks.*

[*http://www.ins.gov.co/Direcciones/Vigilancia/Lineamientosydocumentos/Lineamientos%202019.pdf*](http://www.ins.gov.co/Direcciones/Vigilancia/Lineamientosydocumentos/Lineamientos%202019.pdf)

Line 306: I could not see these discrepancies discussed in the results but may have missed it

*This was misleading and has been edited*

Line 319: What are the recommended first line antibiotics for typhoid? Are there national guidelines?

*Treatment guidelines for typhoid are not standardized in Colombia, the treatment of a patient with suspect of typhoid is usually with third generation cephalosporin. This has been commented on.*

 Line 320: I could not see any results that confirmed that the extended spectrum cephalosporin resistant strain had an ESBL phenotype. It looks as if this strain was negative by PCR for blaSHV, blaTEM, and blaCTX-M?

*This was checked and found to be likely contamination. Three organisms were PCR positive for an ESBL gene but remained susceptible to third generation cephalosporins*

Please explain some of the variables (perhaps as a footnote): Regime type; Categories of health coverage; municipal centre and populated centre.

*These have been added as table footnote*

Reviewer #2: In the spirit of being helpful, the following typographical and grammatical edits are offered:

*Many thanks for these additional edits*

• Line 56: A period is missing at the end of the first sentence

*This has been added*

• Line 59: “we aimed to add some insight” is informal

*This has been edited*

• Line 62: “widely is” should be changed to “is widely”

*This has been edited*

• Line 63: “major” should be written as “majority”

*This has been edited*

• Line 64: “epidemiology of typhoid of Colombia” should be rewritten to “epidemiology of typhoid in Colombia” or similar

*This has been edited*

• Line 64: “distinct to” should be written as “distinct from”

*This has been edited*

• Line 65: “organisms that circulating nationally” should be written as “organisms that are circulating nationally” or “organisms that circulate nationally”

*This has been edited*

• Line 83: misplaced comma between “disease, which”

*This has been edited*

• Line 302: Run-on sentence between “transmission, similar”

*This has been edited*

• Line 303: Missing articles; “currently classified as country with intermediate burden” should include “a” so it reads “as a country” and “an” so it reads “with an intermediate burden”

*This has been edited*

• Line 341: Change 5th word “the” to “that”

*This has been edited*

• Line 360: run-on sentence between “system(s)” and “the data”

*This has been edited*
--------------------

**Summary and General Comments**

Reviewer #1: Typhoid fever was a major public health problem in Latin America in past decades but in recent years it has appears to have declined as a problem. This report is a useful reminder that for some countries and populations it remains an important disease. Importantly, unlike other regions of the world, antimicrobial resistance was uncommon and restricted to low levels of resistance to ampicillin and fluoroquinolones. A diversity of genetic types was described and limited overlap with genetic types in other Latin American countries. There was some evidence of areas with a higher incidence.

Detection of cases is dependent on a positive blood or bone marrow culture. It would be helpful to have some more detailed background information about the distribution of laboratories with the capacity for blood culture. Would most patients with a fever been investigated with a blood culture? In particular would blood cultures be performed in children and in smaller hospitals outside the main department centres or is it possible that there is under-ascertainment of cases in these groups?

*We have included some information explain the distribution of blood culturing labs throughout the country.*

Reviewer #2: The manuscript reports that over a 4-year period (2012-2015) in Colombia the national public health surveillance protocol for the identification of typhoid and paratyphoid fever yielded 468 confirmed cases of typhoid fever that had an archived Salmonella Typhi isolate. Notably, 420 of the 468 isolates originated from seven departments of Colombia, with 190/420 isolates coming from a single department, Norte de Santander, which borders Venezuela. Most cases occurred in the main metropolitan centers in the affected departments and 68.2% of aces were in persons > 15 years of age. Importantly, the vast majority of the isolates were sensitive to first-line antibiotics and none were H58 lineage. Pulse field gel electrophoresis (PFGE) was performed on 402 S. Typhi isolates allowing special distribution analyses to be carried out. These data shed light on some geographic areas and sub-populations in Colombia where typhoid fever remains a public health problem. These data do provide helpful information on typhoid fever in Colombia. Whilst the PFGE patterns if Colombian isolates are useful for analyzing relatedness among strains from within the same department and between different departments of Colombia, the attempt to relate the PFGE patterns to isolates from other Latin American countries was not particularly helpful because the source of the strains from other countries was not described in any detail.

*More information regarding these isolates has been added*

Need a table that shows population, cases and incidence per 100,000 for the seven departments and for Cucutá”, the capital city of Norte de Santander department.

*Table S2 included*

This city has been an epicenter of Venezuelan migration. The isolates from Cucutá should be compared to Venezuelan isolates of S. Typhi, if the authors have some.

*Unfortunately, this was not possible*

Our work highlights a substantial burden of typhoid in Colombia, characterized by sustained transmission in some regions and limited epidemics in other departments. This manuscript provides a unique insight into this forgotten disease in Latin America and will pave the wave for further investigations into this disease on the continent.

Yours sincerely,

**Professor Stephen Baker BSc DIC PhD**

Director of Research for Global Health

Cambridge Institute of Therapeutic Immunology & Infectious Disease (CITIID)

Cambridge Biomedical Campus

University of Cambridge

Cambridge, CB2 0AW
